# Interaction Potential of the Multitargeted Receptor Tyrosine Kinase Inhibitor Dovitinib with Drug Transporters and Drug Metabolising Enzymes Assessed *in Vitro*

**DOI:** 10.3390/pharmaceutics6040632

**Published:** 2014-12-16

**Authors:** Johanna Weiss, Dirk Theile, Zdenek Dvorak, Walter Emil Haefeli

**Affiliations:** 1 Department of Clinical Pharmacology and Pharmacoepidemiology, University of Heidelberg, Im Neuenheimer Feld 410, 69120 Heidelberg, Germany; E-Mails: dirk.theile@med.uni-heidelberg.de (D.T.); walter.emil.haefeli@med.uni-heidelberg.de (W.E.H.); 2 Department of Cell Biology and Genetics, Faculty of Science, Palacky University, Slechitelu 11, 78371 Olmouc, Czech Republic; E-Mail: moulin@email.cz

**Keywords:** dovitinib, tyrosine kinase inhibitor, CYPs, drug transporters, drug–drug interaction

## Abstract

Dovitinib (TKI-258) is under development for the treatment of diverse cancer entities. No published information on its pharmacokinetic drug interaction potential is available. Thus, we assessed its interaction with important drug metabolising enzymes and drug transporters and its efficacy in multidrug resistant cells *in vitro*. P-glycoprotein (P-gp, *MDR1*, *ABCB1*) inhibition was evaluated by calcein assay, inhibition of breast cancer resistance protein (BCRP, *ABCG2*) by pheophorbide A efflux, and inhibition of organic anion transporting polypeptides (OATPs) by 8-fluorescein-cAMP uptake. Inhibition of cytochrome P450 3A4, 2C19, and 2D6 was assessed by using commercial kits. Induction of transporters and enzymes was quantified by real-time RT-PCR. Possible aryl hydrocarbon receptor (AhR) activating properties were assessed by a reporter gene assay. Substrate characteristics were evaluated by growth inhibition assays in cells over-expressing P-gp or BCRP. Dovitinib weakly inhibited CYP2C19, CYP3A4, P-gp and OATPs. The strongest inhibition was observed for BCRP (IC_50_ = 10.3 ± 4.5 μM). Among the genes investigated, dovitinib only induced mRNA expression of *CYP1A1*, *CYP1A2*, *ABCC3* (coding for multidrug resistance-associated protein 3), and *ABCG2* and suppressed mRNA expression of some transporters and drug metabolising enzymes. AhR reporter gene assay demonstrated that dovitinib is an activator of this nuclear receptor. Dovitinib retained its efficacy in cell lines over-expressing P-gp or BCRP. Our analysis indicates that dovitinib will most likely retain its efficacy in tumours over-expressing P-gp or BCRP and gives first evidence that dovitinib might act as a perpetrator drug in pharmacokinetic drug–drug interactions.

## 1. Introduction

Dovitinib (TKI-258) is an inhibitor of fibroblast growth factor receptors 1–3 (FGFR), vascular endothelial growth factor receptors 1–3 (VEGFR), platelet-derived growth factor receptor (PDGFR), and other class III receptor tyrosine kinases [1]. It is under development for the treatment of several neoplasias like renal cell carcinoma, advanced breast cancer, gastrointestinal stromal tumours, and hepatocellular carcinoma [2,3,4,5,6,7,8,9,10].

Chemotherapeutic efficacy is often hampered in neoplasias exhibiting multidrug-resistance (MDR), for which one major cause is the over-expression of ATP-binding cassette (ABC)-transporters like P-glycoprotein (P-gp, encoded by *MDR1*, *ABCB1*) or breast cancer resistance protein (BCRP, encoded by *ABCG2*) [11]. Moreover, safety and effectiveness of cytostatic drugs can be altered by drug–drug interactions. Inhibition and induction of enzymes and drug transporters involved in the distribution and metabolism of drugs may critically reduce or increase exposure with drugs, leading to toxic side effects or non-response. Dovitinib is mainly metabolised by flavine monooxygenase and cytochrome P450 (CYP)1A and 3A4 [12]. Due to auto-induction of CYP1A, the exposure with dovitinib at steady-state is less than or equal to that of a single dose [12]. Beyond this, there is no published data on the interaction of dovitinib with drug metabolising enzymes and drug transporters and there is no data at all whether dovitinib retains its efficacy in MDR cells. We therefore investigated (1) whether it inhibits the most important drug transporters modulating intestinal absorption or hepatic uptake (P-gp, BCRP, organic anion transporting polypeptides 1B1 and 1B3 (OATP1B1, OATP1B3)), (2) whether it inhibits important CYPs (CYP3A4, CYP2C19, and CYP2D6), (3) whether it retains its efficacy in MDR cell lines, and (4) whether dovitinib can induce the expression of relevant human drug transporters and drug metabolising enzymes.

## 2. Materials and Methods

### 2.1. Materials

Culture media, medium supplements, Hanks’ balanced salt solution, phosphate-buffered saline (PBS), and anti-β-actin (Clone AC-74) were purchased from Sigma–Aldrich (Taufkirchen, Germany). Foetal calf serum (FCS) and G418 were purchased from PAA (Pasching, Austria). Crystal violet, dimethyl sulfoxide (DMSO), TRIS (2-amino-2-(hydroxymethyl)-propan-1,3-diol), sodium dodecyl sulphate (SDS), glycerol, Tween^®^20, dithiothreitol (DTT), rifampicin, Triton^®^ X-100, and sodium dodecyl sulphate (SDS) from AppliChem (Darmstadt, Germany). Calcein acetoxymethylester (calcein-AM) was obtained from Invitrogen (Karlsruhe, Germany) and pheophorbide A from Frontier Scientific Europe (Carnforth, UK). 8-fluorescein-cAMP was obtained from BIOLOG Life Science Institute (Bremen, Germany), 2,3,7,8-tetrachlordibenzo-*p*-dioxin (TCDD, dioxin) was obtained from LGC Standards GmbH (Wesel, Germany). Nitrocellulose membranes (Optitran BA-S 85) were obtained from Schleicher & Schuell BioScience (Dassel, Germany). The antibody against human P-gp clone C219 was obtained from Calbiochem (Darmstadt, Germany). Cell culturing bottles were supplied by Greiner (Frickenhausen, Germany) and 96-well microtiter plates by Nunc (Wiesbaden, Germany). White 96-well plates with clear bottom and clear lid for luminometry were supplied by Greiner (Frickenhausen, Germany). RNeasy Mini-Kit was purchased from Qiagen (Hilden, Germany). The RevertAid™ H Minus First Strand cDNA Synthesis Kit was obtained from Fermentas (St. Leon-Rot, Germany). LC480 SYBR Green I Master and TaqMan Gene Expression MasterMix were supplied by Roche Applied Science (Mannheim, Germany), Absolute QPCR SYBR Green Mix by Abgene (Hamburg, Germany), the QuantiTect^®^ Primer Assay by Qiagen (Hilden, Germany), and the TaqMan^®^ Gene Expression Assay by Applied Biosystems (Carlsbad, CA, USA). The Steady-Glo™ Luciferase Assay System was obtained from Promega Corporation (Madison, WI, USA). Dovitinib was purchased from Sequoia Research Products (Pangbourne, UK).

### 2.2. Stock Solutions

Stock solutions of dovitinib (10 mM, highest possible solution) and rifampicin (100 mM) were prepared in DMSO. Due to limited solubility in aqueous media, dovitinib could only be tested up to 100 µM in the calcein assay, up to 100 µM in the pheophorbide A assay, the 8-fluorescein-cAMP assay, and the growth inhibition assays, and up to 20 µM in the CYP inhibition assays.

### 2.3. Cell Lines

#### 2.3.1. LS180 Cells

The human colon adenocarcinoma cell line LS180 (available at ATCC, Manassas, VA, USA) was used for induction experiments as a surrogate for the intestine being a major site of drug interactions and being an ideal model for investigating pregnane X receptor (PXR) and aryl hydrocarbon receptor (AhR) mediated induction [13,14,15,16,17,18,19,20]. Cells were cultured under standard cell culture conditions with Dulbecco’s Modified Eagle’s Medium (DMEM) supplemented with 10% fetal calf serum, 2 mM glutamine, 100 U/mL penicillin, 100 µg/mL streptomycin sulphate, and 0.1 mM nonessential amino acids.

#### 2.3.2. MDCKII Cells

The transporter over-expressing cell lines MDCKII-MDR1 [21] and MDCKII-BCRP [22] and the corresponding parental cell line MDCKII (Madin–Darby canine kidney cells) were used to assess the sensitivity of cells with and without P-glycoprotein (P-gp/*ABCB1*), or breast cancer resistance protein (BCRP/*ABCG2*) expression as an indirect marker for dovitinib substrate properties of the respective transporter. Moreover, MDCKII-BCRP cells were used to assess BCRP inhibition by dovitinib. MDCKII over-expressing cell lines were kindly provided by Dr. A.H. Schinkel and Dr. P. Borst (The Netherlands Cancer Institute, Division of Experimental Therapy, Amsterdam, The Netherlands). The parental cell line MDCKII (available at ATCC, Manassas, VA, USA) was used as a control. The cells were cultured in DMEM containing 10% FCS, 2 mM glutamine, 100 U/mL penicillin, and 100 µg/mL streptomycin sulphate.

#### 2.3.3. LLC-PK1 and L-MDR1 Cells

L-MDR1 cells, a porcine kidney epithelial cell line over-expressing the human *MDR1/ABCB1* gene [23] and the corresponding parental cell line LLC-PK1 (available from ATCC, Manassas, VA, USA) were used for P-gp inhibition assays as a control. The L-MDR1 cell line was kindly provided by Dr. A.H. Schinkel (The Netherlands Cancer Institute, Division of Experimental Therapy, Amsterdam, The Netherlands). The cells were cultured under standard cell culture conditions with medium M199 supplemented with 10% FCS, 2 mM glutamine, 100 U/mL penicillin, and 100 µg/mL streptomycin sulphate. To maintain P-gp/*ABCB1* expression the culture medium for L-MDR1 was supplemented with 0.64 µM vincristine. One day before the P-gp inhibitiion assay, both cell lines were fed with vincristine-free culture medium.

#### 2.3.4. HEK293 Cells

For assessing human organic antion transporting polypeptides (OATPs = solute carriers of organic anions (SLCOs)) inhibition, the human embryonic kidney cell line HEK293 stably transfected with OATP1B1 (HEK-OATP1B1), OATP1B3 (HEK-OATP1B3), or the empty control vector (HEK293-VC G418) were used [24,25]. Cells were cultured under standard cell culture conditions with DMEM supplemented with 10% FCS, 2 mM glutamine, 100 U/mL penicillin, 100 µg/mL streptomycin sulphate, and 800 µg/mL G418. Cells were kindly provided by Dr. D. Keppler (German Cancer Research Centre, Heidelberg, Germany).

#### 2.3.5. AZ-AHR Cells

Human hepatoma HepG2 cells stably transfected with a construct containing several AhR binding sites upstream of a luciferase reporter gene were used for the AhR reporter gene assays as described previously [26].

### 2.4. Cytotoxicity Assay

Dovitinib was tested for cytotoxic effects prior to P-gp, BCRP, and OATP inhibition assays with the Cytotoxicity Detection Kit (Roche Applied Science, Mannheim, Germany) according to the manufacturer’s instruction. Dovitinib was not cytotoxic up to 100 µM in all cell lines tested.

### 2.5. P-gp Inhibition Assay (Calcein Uptake Assay)

The calcein assay was used to assess P-gp inhibition in L-MDR1 cells. Calcein-AM is a fluorogenic, highly lipid-soluble dye which rapidly permeates the plasma membrane. Endogenous esterases cleave the ester bonds intracellularly, producing the hydrophilic and fluorescent dye calcein, which cannot leave the cell via the plasma membrane [27]. Cells expressing high levels of P-gp rapidly extrude nonfluorescent calcein-AM from the plasma membrane, thus preventing accumulation of fluorescent calcein in the cytosol. Thus, inhibition of P-gp will lead to intracellular calcein accumulation. Each concentration (0.1–100 µM) was tested in octuplet and each experiment was performed at least in triplicate.

For calculation of the inhibitory effects in the calcein assay, the f2-value (concentration needed to increase baseline fluorescence by factor 2 ± standard deviation) was calculated as described and validated previously [28,29], because dovitinib did not reach plateau effects making the calculation of an IC_50_ value impossible.

### 2.6. BCRP Inhibition Assay (Pheophorbide A Flow Cytometry Efflux Assay)

Flow cytometric BCRP inhibition assays with MDCKII and over-expressing MDCKII-BCRP cells and pheophorbide A as a fluorescent BCRP substrate were conducted as described and validated previously [30]. In brief, cells were suspended in incubation medium (RPMI with 2% FCS) containing 1 µM pheophorbide A and incubated at 37 °C for 30 min on a rotary shaker (450 rpm). Cells were then washed once with ice-cold incubation medium and resuspended in 500 incubation medium containing dovitinib at various concentrations (0.1–100 µM). After incubation for 60 min at 37 °C on a rotary shaker, cells were washed with ice-cold PBS with 2% FCS, resuspended in ice-cold PBS with 2% FCS and kept on ice until flow cytometry. Intracellular fluorescence was analysed in a Becton Dickinson LSR II flow cytometer with a solid state coherent sapphire blue laser and a 660 bandpass filter for pheophorbide A.

In each sample 30,000 cells were counted. Cell debris was eliminated by gating the living cells in the forward *versus* side scatter. BCRP positive MDCKII-BCRP cells were additionally gated using their GFP-signal. To quantify the inhibitory effects of the compounds, the ratio between the median fluorescence with inhibitor and without inhibitor during the efflux period was calculated and normalised to the effect observed in the corresponding parental cell line. Each experiment was performed in triplicate.

### 2.7. OATP Inhibition Assay (8-FcA Flow Cytometry Uptake Assay)

Inhibition of OATP1B1 and OATP1B3 was analysed by flow cytometric uptake of the fluorescent 8-FcA as described previously [13] using HEK-OATP1B1, OATP1B3, and HEK293-VC G418 (as a control). In brief, cells were washed once in 500 µL incubation medium (PBS with 2% FCS) and subsequently incubated with 100 µL incubation medium containing 2.5 µM 8-FcA with or without dovitinib (0.05–100 µM) under light protection at 37 °C for 10 min on a rotary shaker (450 rpm). Subsequently, cells were washed once with 1 mL ice-cold incubation medium and resuspended in 500 µL incubation medium and kept on ice until flow cytometry. Intracellular fluorescence was analysed in a Becton Dickinson LSRII flow cytometer (Becton Dickinson, Heidelberg, Germany) with a solid state coherent sapphire blue laser and a 530 bandpass filter for 8-FcA.

In each sample 30,000 cells were counted. Cell debris was eliminated by gating the viable cells in the forward *versus* side scatter. For calculation of the inhibitor effects, the ratio between the median fluorescence (MF) of intracellular 8-FcA with and without inhibitor was calculated. The effect in the cell line HEK293-VC G418 was used to check whether the effects observed can be attributed to OATP inhibition. Each experiment was performed at least in triplicate.

### 2.8. Inhibition of CYP3A4, CYP2C19, and CYP2D6

Inhibition studies were performed with the CYP2D6/AMMC, the CYP2C19/CEC, and the CYP3A4/BFC High Throughput Inhibitor Screening Kit (Becton Dickinson Biosciences, Heidelberg, Germany) according to the manufacturer’s instructions. The Screening Kits contain the respective recombinant CYP and fluorogenic substrates, which are blocked dyes yielding minimal fluorescence signal until cleaved by the enzyme. Dovitinib was analysed for its capacity to inhibit the production of the fluorescent signal. Eight concentrations (9 nM–20 µM) were tested and the experiment was conducted in triplicate. Omeprazole served as a control compound for CYP2C19 inhibition, quinidine for CYP2D6, and ketoconazole for CYP3A4.

### 2.9. Growth Inhibition Assay

Growth inhibition assays in LS180 cells were conducted to determine suitable maximum concentrations for the induction assay without profound antiproliferative effects. Proliferation was quantified by crystal violet staining as described previously [31]. In brief, a 100 µL aliquot of each cell suspension at a concentration of 3 × 10^5^ cells/mL was seeded onto collagen-coated 96-well microtiter plates and incubated for 24 h. Medium was substituted for dovitinib-containing medium and the cells were incubated for further 48 h. Subsequently, cells were washed once with PBS and viable adherent cells were stained with 50 µL of 0.5% (*w*/*v*) crystal violet in 20% methanol in aqua bidest per well for 15 min. After staining, plates were washed with aqua bidest and dried for 4 h in a drying chamber at 37 °C. To dissolve crystal violet, 200 µL of methanol were added to each well. Absorption was measured at 555 nm excitation. Proliferation was expressed as proliferation index, which was calculated as the absorption intensity of the test well in percentage points relative to zero proliferation (absorption of wells containing only medium set to 0%) and native proliferation (absorption intensity of untreated control cells set to 100%). Each experiment was performed at least in triplicate with *n* = 8 wells for each concentration. Concentration-response curves and IC_20_ values were calculated by GraphPad Prism version 5.02 (GraphPad Software Inc., La Jolla, CA, USA) according to a sigmoid *E*_max_ model. The IC_20_ for proliferation inhibition by dovitinib was 4.9 ± 2.5 µM, thus the maximum concentration for the induction assay was set to 5 µM ensuring at least 20% of the cells survive.

Moreover, growth inhibition assays in MDCKII, and their ABC-transporter over-expressing counterparts (MDCKII-MDR1, MDCKII-BCRP) were conducted to evaluate whether dovitinib sustains its efficacy in multidrug resistant cell lines.

### 2.10. Induction Assay

The human colon adenocarcinoma cell line LS180 (available at ATCC, Manassas, VA, USA) was used for induction experiments as a surrogate for the intestine being a major site of drug interactions and being an ideal model for investigating PXR and AhR mediated induction [11,16,17,18,19,20,21,22]. Cells were cultured as described previously [18]. For the induction experiments, LS180 cells were seeded in 75 cm^2^ culturing flasks and incubated for three days. Cells were then treated with culture medium containing dovitinib (0.1–5 µM) in quadruplicate for 4 consecutive days. Rifampicin (20 µM) served as a positive control and culture medium with 0.02% DMSO as a negative control. For induction of CYP1A1 and CYP1A2, 5 nM TCDD was used as a positive control. All incubation solutions were adjusted to 0.02% DMSO. After harvesting, cells were splitted for RNA and protein extraction.

### 2.11. Quantification of mRNA Expression by Real-Time RT-PCR

RNA was isolated using the RNeasy Mini-Kit and cDNA was synthesised with the RevertAid™ H Minus First Strand cDNA Synthesis Kit according to the manufacturer’s instructions. mRNA expression was quantified by real-time RT-PCR with the LightCycler^®^ 480 (Roche Applied Science, Mannheim, Germany) as described previously [16,32]. Primer sequences were published previously [16,33], CYP1A1 [34], CYP1A2 [35]. The most suitable housekeeping gene for normalisation in LS180 cells was identified using geNorm (version 3.4, Center for Medical Genetics, Ghent, Belgium), which determines most stable reference genes from a set of tested genes in a given cDNA sample panel [36]. Among a panel of eight housekeeping genes tested, glucose-6-phosphate dehydrogenase (*G6PDH*) was the most stable housekeeping gene in LS180 cells under our experimental conditions. Data were evaluated by calibrator-normalised relative quantification with efficiency correction using the LightCycler^®^ 480 software version 1.5 (Roche Applied Science, Mannheim, Germany). The software calculated the relative amount of the target gene and the reference gene (*G6PDH*) based on the crossing points. Results were expressed as the target/reference ratio divided by the target/reference ratio of the calibrator. The results are therefore corrected for sample in homogeneities and variance caused by detection. All samples were amplified in duplicate. The following target genes were quantified: *CYP1A1*, *CYP1A2*, *CYP2C19*, *CYP3A4*, *ABCB1*, *ABCC1* (coding for multidrug resistance-associated protein 1, MRP1), *ABCC2*, *ABCC3*, *ABCG2*, *SLCO1B1* (coding for OATP1B1), *SLCO2B1*, *UDP-glucuronosyltransferase 1A3 (UGT1A3)*, *UGT2B7*, and *NR1I2* (coding for the PXR).

### 2.12. Western Blot Analysis of P-gp Expression

P-gp protein expression was analysed in triplicate by SDS polyacrylamide gel electrophoresis (SDS-PAGE) and western blotting. In brief, cell lysates containing 20 µg protein were mixed with 5× sample buffer (containing Tris–HCl, SDS, DTT, bromphenolblue, and glycerol) and subjected to a 10% SDS-PAGE. After electrophoresis, proteins were electrotransferred to nitrocellulose nitrate membranes. Blots were blocked by incubation for 20–40 min with 5% skim milk (*w*/*v*) in phosphate-buffered saline containing 0.1% Tween^®^20. Protein detection was carried out with murine monoclonal antibodies raised against human P-gp (diluted 1:100 in Tris-buffered saline containing 0.1% Tween^®^20 (TBST)) or β-actin (Clone AC-74; diluted 1:40,000). After extensive washing of the membranes, blots were incubated with horseradish peroxidase-linked secondary anti-mouse antibody (Amersham, Freiburg, Germany). Bands were visualised by enhanced chemiluminescence using the SuperSignal^®^West Pico Chemiluminescent Substrate Kit (Pierce, Rockford, IL, USA).

### 2.13. AhR Reporter Gene Assay

An AhR reporter gene assay was applied to investigate whether dovitinib is an AhR activator. In total, 60,000 AZ-AhR cells were seeded into each well of 96-well plates with clear bottom and clear lid for luminometry. After 24 h of incubation, cells were treated with TCDD (positive control, 0.1–50 nM), dovitinib (5–5000 nM) or vehicle control (0.02% DMSO) for further 24 h. The assay was performed with the Steady-Glo™ Luciferase Assay System according to the manufacturer’s instructions. Drug-induced increases of AhR receptor activity were normalised to activity of non-drug treated controls set to 1 (=100%). EC_50_ value is expressed as mean ± S.D. for *n* = 3 experiments with triplicates for each concentration tested.

### 2.14. Statistical Analysis

Data were analysed using GraphPad Prism Version 5.02 and InStat Version 3.06 (GraphPad Software, San Diego, CA, USA). The differences in mRNA expression following incubation with the compounds investigated compared with the respective vehicle controls were tested using ANOVA with Dunnett’s *post hoc* test or with student’s two-tailed *t*-test (TCDD *versus* control). The differences of the IC_50_ values obtained in the growth inhibition assays were tested using two-tailed *t*-test. *p* ≤ 0.05 was considered significant.

## 3. Results

### 3.1. Dovtinib Weakly Inhibits P-gp

**Figure 1:**
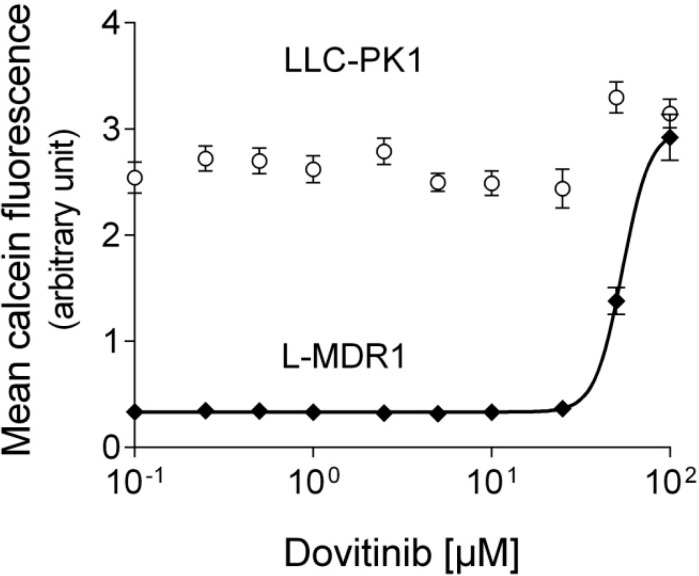
Calcein assay. Concentration-dependent effect of dovitinib on baseline calcein fluorescence in L-MDR1 cells and lack of effect in the corresponding parental cell line LLC-PK1 indicating P-gp inhibition by dovitinib. Each curve depicts one representative experiment of a series of 3. Data are expressed as mean ± SEM for *n* = 8 wells.

In contrast to LLC-PK1, L-MDR1 cells exhibit very low intracellular calcein fluorescence confirming the efflux activity of P-gp. Dovitinib (tested from 0.1 to 100 µM) increased intracellular calcein fluorescence at higher concentrations in L-MDR1 cells but not in the parental cell line indicating weak concentration-dependent P-gp inhibition (Figure 1, Table 1).

**Table 1:** IC_50_ and f2 (concentration needed to increase baseline fluorescence two-fold) values for transporter inhibition assays for dovitinib and the corresponding control compound.

Protein Inhibited	Cell Model Used	Dovitinib f2 [µM]	Control Compound	f2 [µM]
P-gp	L-MDR1	48.4 ± 15.6	Verapamil	3.9 ± 1.9 [28]
		**IC_50_ [µM]**		**IC_50_ [µM]**
BCRP	MDCKII-BCRP	10.3 ± 4.5	Fumitremorgin C	0.7 ± 0.3 [37]
OATP1B1	HEK-OATP1B1	76.5 ± 28.9	Rifampicin	2.4 ± 0.9 [13]
OATP1B3	HEK-OATP1B3	105.2 ± 23.0	Rifampicin	2.1 ± 1.0 [13]

### 3.2. Dovitinib Weakly Inhibits OATP1B1 and OATP1B3

8-Fluorescein-cAMP is a prototype substrate of OATP1B1 and OATP1B3 uptake transporters. Dovitinib (tested from 0.05 to 100 µM) concentration-dependently inhibited 8-fluorescein-cAMP uptake at higher concentrations in HEK-OATP1B1 and HEK-OATP1B3 cells but not into the control cell line HEK293-VC G418. Compared to the IC_50_ values of rifampicin (inhibitor positive control), OATP1B1 and OATP1B3 inhibition by dovitinib was rather weak (Table 1).

### 3.3. Dovitinib Inhibits BCRP

MDCKII cells overexpressing BCRP (MDCKII-BCRP) hardly accumulate pheophorbide A due to active efflux of the fluorescent compound. Thus, enhanced accumulation in these cells indicates drug transporter inhibition. Dovitinib (tested from 0.1 to 100 µM) concentration-dependently increased intracellular pheophorbide A fluorescence in MDCKII-BCRP cells but not in the parental cell line lacking human BCRP) indicating BCRP inhibition. This inhibition was stronger than for the other transporters tested being in the lower micromolar range (Table 1).

### 3.4. Inhibition of CYPs

For CYP inhibition, IC_50_-values could not be calculated, because dovitinib did not reach 100% inhibition up to 20 µM. It only weakly inhibited CYP2C19 (about 50% at 20 µM) and CYP3A4 (about 30% at 20 µM), whereas CYP2D6 was not inhibited at all. Corresponding IC_50_ values for the control compounds were for omeprazole (CYP2C19) 0.8 ± 0.2 µM, for quinidine (CYP2D6) 5.9 ± 2.5 nM, and for ketoconazole (CYP3A4) 35.3 ± 14.6 nM.

### 3.5. Influence of Dovitinib on the mRNA Expression of Drug Transporters, Drug Metabolising Enzymes, and PXR

**Figure 2:**
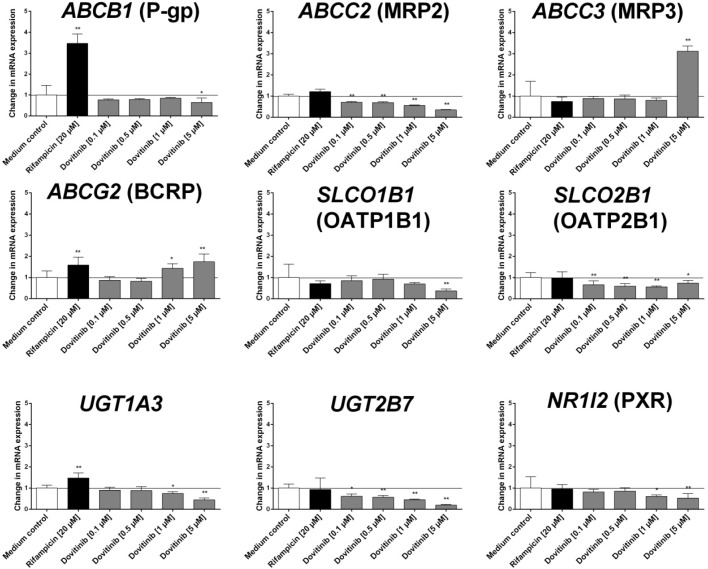
Induction assay. Concentration-dependent effect of dovitinib (0.1–5 µM) and 20 µM rifampicin (positive control) after four days on mRNA expression in LS180 cells compared to untreated medium control. Expression data were normalised to the housekeeping gene *G6PDH*. Data are expressed as mean ± SEM for *n* = 8 (four biological replicates and two PCR runs for every sample) and normalised to medium control (set to 1). The differences in mRNA expression following incubation with the compounds investigated compared with the respective vehicle control were tested using ANOVA with Dunnett’s *post hoc* test. * *p* < 0.05, ** *p* < 0.01.

**Figure 3:**
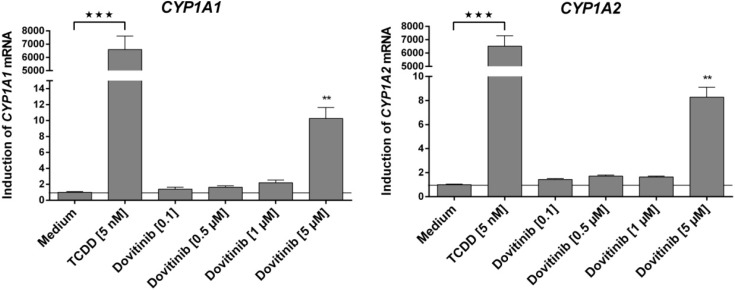
Induction assay. Concentration-dependent effect of dovitinib (0.1–5 µM) and 5 nM TCDD after four days on mRNA expression in LS180 cells. Expression data were normalised to the housekeeping gene *G6PDH*. Data are expressed as mean ± SEM for *n* = 18 (four biological replicates and two PCR runs for every sample) and normalised to medium control (set to 1). Statistical significance was tested with student’s two-tailed *t*-test for TCDD compared to medium control and with ANOVA with Dunnett’s *post hoc* test (for dovitinib compared to medium control, TCDD excluded). ** *p* < 0.01, *** *p* < 0.01.

Dovitinib (tested from 0.1 to 5 µM) only induced mRNA expression of *CYP1A1*, *CYP1A2*, *ABCC3* and *ABCG2* (Figure 2 and Figure 3). In contrast, mRNA expression of *ABCB1*, *ABCC2*, *SLCO1B1*, *SLCO2B1*, *UGT1A3*, *UGT2B7*, and *NR1I2* was even reduced at higher concentrations of dovitinib (Figure 2). Dovitinib had no influence on the mRNA expression of *CYP2C19*, *CYP3A4* and *ABCC1*.

**Figure 4:**
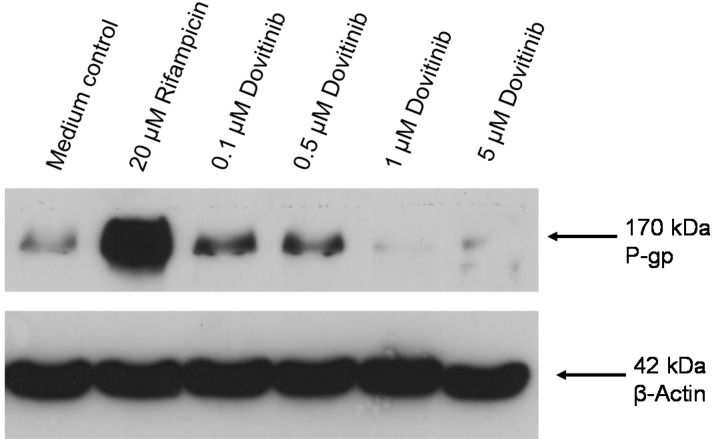
Western blot. Concentration-dependent effect of dovitinib (0.1–5 µM) and 20 µM rifampicin (positive control) after four days on P-gp protein expression in LS180 cells compared to untreated medium control. β-actin served as a loading control. Depicted is one blot of a series of three.

Repression of P-gp expression at higher dovitinib concentrations was verified at the protein level. Western blot analysis clearly demonstrated suppression of P-gp protein expression, which was even more pronounced than at the mRNA level, whereas a small increase in P-gp expression was observed at 0.1 and 0.5 µM dovitinib (Figure 4).

### 3.6. Activation of AhR Activity

**Figure 5:**
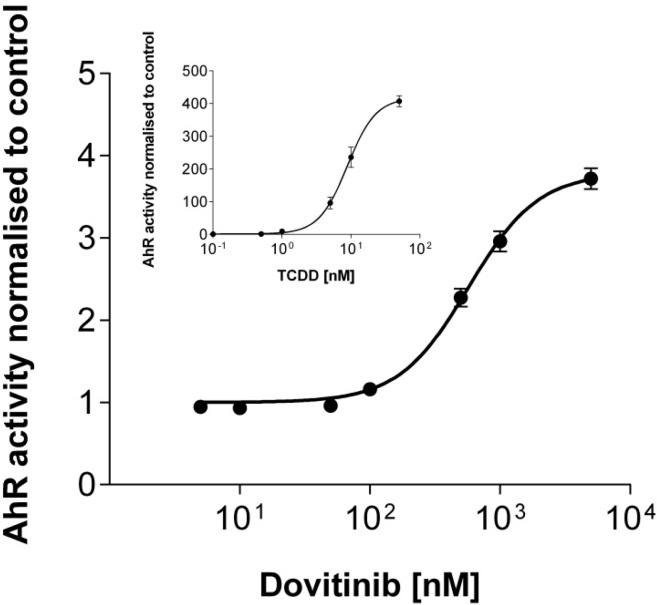
Reporter gene assay. Concentration-dependent effect of dovitinib (0.005–5 µM) and TCDD (0.01–50 nM; insert) on AhR activity in AZ-AhR cells. Each curve depicts the results of three experiments with each concentration tested in triplicate. Data are expressed as mean ± SEM for *n* = 9.

Dovitinib clearly activated AhR activity in a concentration-dependent manner with an EC_50_ value of 609 ± 282 nM (Figure 5).

### 3.7. Efficacy in MDR Cell Lines

**Figure 6:**
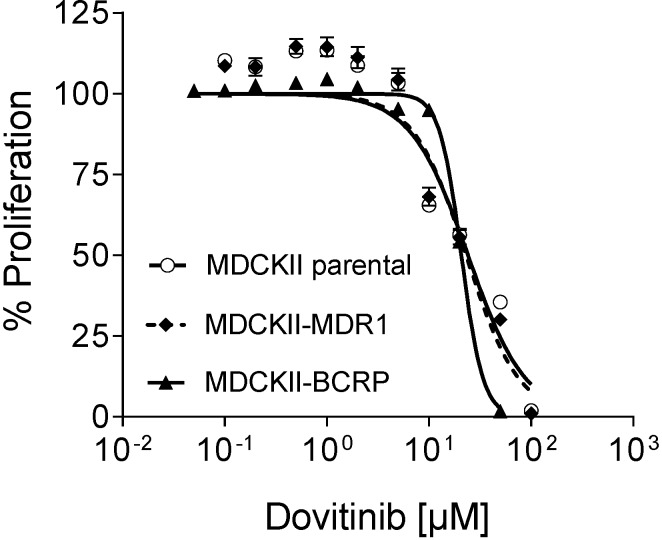
Growth inhibition assay. Concentration-dependent effect of dovitinib (0.1–100 µM) on the proliferation of the P-gp over-expressing cell line MDCKII-MDR1, the BCRP over-expression cell line MDCKII-BCRP, and the control cell line MDCKII parental. Each curve depicts the results of four experiments with each concentration tested in octuplet. Data are expressed as mean ± SEM for *n* = 32 wells.

Growth inhibition assays in MDCKII, MDCKII-BCRP, and MDCKII-MDR1 cells consistently demonstrated that the cells over-expressing efflux transporters are not more resistant towards antiproliferative effects of dovitinib (tested from 0.05 to 100 µM) than the native cell line indicating that dovitinib is not transported by these transporters (MDCKII, IC_50_ = 20.0 ± 2.9 µM; MDCKII-BCRP, IC_50_ = 21.4 ± 3.6 µM; MDCKII-MDR1, IC_50_ = 18.3 ± 4.1 µM) (Figure 6).

## 4. Discussion

Tyrosine kinase inhibitors represent a comparatively new and promising class of antineoplastic agents. However, their clinical efficacy is often restricted due to rapid development of drug resistance of the tumour cells. Moreover, interaction with concomitantly used drugs can impair their therapeutic effect and safety [38].

Dovitinib, a new tyrosine kinase inhibitor targeting multiple receptors, is currently under clinical development and has a unique inhibition and acceptable safety profile [39,40]. So far, there is no published data on the interaction profile of dovitinib, on its effects on drug transporters, and on its efficacy in multidrug resistant cell lines. Our data indicate that dovitinib possesses a low interaction potential and might not be prone to reduced efficacy in P-gp or BCRP overexpressing tumours.

In contrast to all approved tyrosine kinase inhibitors, which are substrates of the ABC-transporters P-glycoprotein (P-gp, *ABCB1*) and breast cancer resistance protein (BCRP, *ABCG2*) [30], our data indicate that dovitinib retains its efficacy in multidrug resistant cell lines over-expressing P-gp or BCRP due to absent efflux by these proteins. Moreover, dovitinib does not induce mRNA expressions of *ABCB1*, *ABCC1* (coding for multidrug resistance-associated protein 1 (MRP1)), and *ABCC2* (MRP2), which are all major efflux transporters conferring multidrug resistance. In contrast, mRNA expressions of *ABCC3* (MRP3) and *ABCG2* are induced, which may be important for the efficacy of chemotherapeutics when these drugs’ intestinal absorption (assuming oral administration) or transport out of the tumor cell is modulated by MRP3 or BCRP, respectively.

After prolonged exposure to dovitinib, the expression of most investigated genes was reduced. While this mostly occurred at rather high concentrations, for important pharmacokinetic target sites such as MRP2 it was also observed at expected therapeutic concentrations [39]. Whether this interferes with MDR or leads to systemic drug–drug interactions with substrates of the corresponding proteins and thus potentially contributes to the overall action of the drug requires further assessment. However, it is already well-documented that suppression of MDR protein expression can contribute to efficacy of antineoplastic (combination-) chemotherapy. Gefinitib has been shown to reverse gemcitabine-mediated induction of MRP1–3 [41]. Mechanistically, this observation is quite comprehensible, because kinases of signal transduction have a relevant impact on drug transporter protein expression and phenotypic MDR through phosphorylation [42,43]. Because dovitinib is a multitargeted kinase inhibitor, we cannot rule out that this pleiotropic inhibition of kinase activity causally led to suppression of drug transporters also in our study. Besides influence of kinase activity, inhibition of the nuclear factor κB signaling pathway has also been shown to reduce the expression of P-gp [44]. Interference with the human nuclear factor 4α-binding activity e.g., via the constitutive androstane receptor (CAR) can additionally reduce the expression of UGT2B7 [45]. So far, it is unknown whether dovitinib interacts with these pathways and whether it is a ligand of nuclear receptors such as CAR. However, the lack of induction of *CYP3A4*, *ABCB1*, and *ABCC2* strongly suggests that dovitinib is no ligand of PXR being the major transcription factor responsible for induction of these particular genes. This clearly distinguishes dovitinib from other tyrosine kinase inhibitors like erlotinib, gefitinib, nilotinib, sorafenib, and vandetanib, which all can transcriptionally enhance mRNA expression of P-gp (*ABCB1)* and are thus supposed to be activators of PXR [46]. In contrast to PXR, another nuclear receptor was clearly activated. Using a reporter gene assay, we demonstrated dovitinib to be an activator of the transcription factor AhR. The activation appears to be small compared to the positive control dioxin. However, comparison of dovitinib to this toxin might lead to underestimation of the effect of dovitinib, since dioxin exerts extraordinarily strong effects which have never been reached by any other drug tested so far. Nevertheless, our finding of AhR activation by dovitinib coherently explains the strong induction (approximately 10-fold) of *CYP1A1* and *CYP1A2* (Figure 3), two genes whose expressions are closely regulated by this protein. It also explains the auto-induction of CYP1A demonstrated *in vivo* [12] and the induction of *ABCG2*, which is also a typical target gene of AhR [47].

In addition to the inductive potential of dovitinib, we also demonstrated that dovitinib is only a weak inhibitor of several CYPs, P-gp, OATP1B1, and OATP1B3 protein functions. Only BCRP was markedly inhibited in the lower micromolar range.

The question always arising when obtaining interaction data *in vitro* is whether drug concentrations in patients are high enough to exert similar effects also *in vivo*. In a dose-escalating trial in patients with advanced solid tumours, maximum plasma concentrations lay between 27 and 206 nM [39], being several orders of magnitude below effective concentrations *in vitro*. Only in the intestine, where local concentrations are much higher (about 1 mM after ingestion of a 125 mg dose, according to a formula published by the Food and Drug Administration [48]), inhibition of transporter function, especially BCRP, and effects on mRNA expression of drug transporters or drug metabolising enzymes, are conceivable. Since auto-induction of CYP1A has been observed clinically, this might also affect other CYP1A substrates and substrates of other genes induced or suppressed by dovitinib in our study. However, only clinical studies will answer the question, whether dovitinib provokes clinically relevant drug–drug interactions.

Limitations: (1) We have analysed a selection of drug transporters and drug metabolising enzymes known to contribute to drug–drug interactions. Other enzymes and transporters might also modulate safety and effectiveness of dovitinib; (2) We only investigated induction in LS180 cells, which, however, are a well-established and widely used model especially for induction via PXR and AhR [14,15,16,17,18,19,20,49]. Gene induction via PXR has been demonstrated for most of the genes investigated [47,50,51,52,53,54,55]. However, the extent of gene induction and the induction profile might be different in other cell lines and LS180 cells have been demonstrated to be inferior when investigating CAR mediated induction [15]; (3) In the majority of cases, variations in mRNA levels are translated into changes of the corresponding protein or altered function [3,33,52,56]. We therefore chose to confirm the mRNA data only for P-gp, *i.e.*, the transporter with the highest significance for chemotherapy resistance; (4) We did not measure dovitinib efflux directly, but indirectly with a proliferation assay. However, if a transporter over-expressing cell line is not more resistant to an antineoplastic agent than the parental cell line (lacking the respective transporter), this is a strong indicator for lack of transport by the corresponding transporter.

## 5. Conclusions

In conclusion our data provide a comprehensive analysis of the *in vitro* interaction profile of dovitinib. So far, all data indicates that dovitinib will most likely retain its efficacy even in tumours over-expressing drug efflux transporters or to even enhance co-administered anti-cancer drugs by suppressing MDR protein expression. Pharmacokinetically, our data gives first evidence that dovitinib might interact as a perpetrator drug with substrates of CYP1A, P-gp, BCRP, MRP1, MRP3, OATP1B1, OATP1B3, and UGT1A3 at pharmacological barriers exposed to higher drug concentrations such as the intestine.

## References

[1] Kim K.B., Saro J., Moschos S.S., Hwu P., Tarhini A.A., Hwu W., Jones G., Wang Y., Rupani H., Kirkwood J.W. (2008). A phase I dose finding and biomarker study of TKI258 (dovitinib lactate) in patients with advanced melanoma. J. Clin. Oncol..

[2] André F., Bachelot T., Campone M., Dalenc F., Perez-Garcia J.M., PHurvitz S.A., Turner N., Rugo H., Smith J.W., Deudon S. (2013). Targeting FGFR with Dovitinib (TKI258): Preclinical and Clinical Data in Breast Cancer. Clin. Cancer Res..

[3] Angevin E., Lopez-Martin J.A., Lin C.C., Gschwend J.E., Harzstark A., Castellano D., Soria J.C., Sen P., Chang J., Shi M. (2013). Phase I study of dovitinib (TKI258); an oral FGFR; VEGFR; and PDGFR inhibitor; in advanced or metastatic renal cell carcinoma. Clin. Cancer Res..

[4] Kang Y.K., Yoo C., Ryoo B.Y., Lee J.J., Tan E., Park I., Park J.H., Choi Y.J., Jo J., Ryu J.S. (2013). Phase II study of dovitinib in patients with metastatic and/or unresectable gastrointestinal stromal tumours after failure of imatinib and sunitinib. Br. J. Cancer.

[5] Konecny G.E., Kolarova T., O’Brien N.A., Winterhoff B., Yang G., Qi J., Qi Z., Venkatesan N., Ayala R., Luo T. (2013). Activity of the fibroblast growth factor receptor inhibitors dovitinib (TKI258) and NVP-BGJ398 in human endometrial cancer cells. Mol. Cancer Ther..

[6] Tai W.T., Cheng A.L., Shiau C.W., Liu C.Y., Ko C.H., Lin M.W., Chen P.J., Chen K.F. (2012). Dovitinib induces apoptosis and overcomes sorafenib resistance in hepatocellular carcinoma through SHP-1-mediated inhibition of STAT3. Mol. Cancer Ther..

[7] Yoo C., Ryu M.H., Na Y.S., Ryoo B.Y., Park S.R., Kang Y.K. (2014). Analysis of serum protein biomarkers, circulating tumor DNA, and dovitinib activity in patients with tyrosine kinase inhibitor-refractory gastrointestinal stromal tumors. Ann. Oncol..

[8] Galsky M.D., Posner M., Holcombe R.F., Lee K.M., Misiukiewicz K., Tsao C.K., Godbold J., Soto R., Gimpel-Tetra K., Lowe N. (2014). Phase Ib study of dovitinib in combination with gemcitabine plus cisplatin or gemcitabine plus carboplatin in patients with advanced solid tumors. Cancer Chemother. Pharmacol..

[9] Escudier B., Grünwald V., Ravaud A., Ou Y.C., Castellano D., Lin C.C., Gschwend J.E., Harzstark A., Beall S., Pirotta N. (2014). Phase II results of Dovitinib (TKI258) in patients with metastatic renal cell cancer. Clin. Cancer Res..

[10] Motzer R.J., Porta C., Vogelzang N.J., Sternberg C.N., Szczylik C., Zolnierek J., Kollmannsberger C., Rha S.Y., Bjarnason G.A., Melichar B. (2014). Dovitinib *versus* sorafenib for third-line targeted treatment of patients with metastatic renal cell carcinoma: An open-label, randomised phase 3 trial. Lancet Oncol..

[11] Gottesman M.M., Fojo T., Bates S.E. (2002). Multidrug resistance in cancer: Role of ATP-dependent transporters. Nat. Rev. Cancer.

[12] Wang X., Kay A., Anak O., Angevin E., Escudier B., Zhou W., Feng Y., Dugan M., Schran H. (2013). Population pharmacokinetic/pharmacodynamic modeling to assist dosing schedule selection for dovitinib. J. Clin. Pharmacol..

[13] Weiss J., Theile D., Spalwisz A., Burhenne J., Riedel K.D., Haefeli W.E. (2013). Influence of sildenafil and tadalafil on the enzyme- and transporter-inducing effects of bosentan and ambrisentan in LS180 cells. Biochem. Pharmacol..

[14] Harmsen S., Koster A.S., Beijnen J.H., Schellens J.H., Meijerman I. (2008). Comparison of two immortalized human cell lines to study nuclear receptor-mediated CYP3A4 induction. Drug Metab. Dispos..

[15] Gupta A., Mugundu G.M., Desai P.B., Thummel K.E., Unadkat J.D. (2008). Intestinal human colon adenocarcinoma cell line LS180 is an excellent model to study pregnane X receptor; but not constitutive androstane receptor; mediated CYP3A4 and multidrug resistance transporter 1 induction: Studies with anti-human immunodeficiency virus protease inhibitors. Drug Metab. Dispos..

[16] Weiss J., Herzog M., Haefeli W.E. (2011). Differential modulation of the expression of important drug metabolising enzymes and transporters by endothelin-1 receptor antagonists ambrisentan and bosentan *in vitro*. Eur. J. Pharmacol..

[17] Brandin H., Viitanen E., Myrberg O., Arvidsson A.K. (2007). Effects of herbal medicinal products and food supplements on induction of CYP1A2; CYP3A4 and MDR1 in the human colon carcinoma cell line LS180. Phytother. Res..

[18] Yamasaki D., Nakamura T., Okamura N., Kokudai M., Inui N., Takeuchi K., Watanabe H., Hirai M., Okumura K., Sakaeda T. (2009). Effects of acid and lactone forms of 3-hydroxy-3-methylglutaryl coenzyme A reductase inhibitors on the induction of MDR1 expression and function in LS180 cells. Eur. J. Pharm. Sci..

[19] Li W., Harper P.A., Tang B.K., Okey A.B. (1998). Regulation of cytochrome P450 enzymes by aryl hydrocarbon receptor in human cells: CYP1A2 expression in the LS180 colon carcinoma cell line after treatment with 2,3,7,8-tetrachlorodibenzo-p-dioxin or 3-methylcholanthrene. Biochem. Pharmacol..

[20] Harper P.A., Prokipcak R.D., Bush L.E., Golas C.L., Okey A.B. (1991). Detection and characterization of the Ah receptor for 2,3,7,8-tetrachlorodibenzo-p-dioxin in the human colon adenocarcinoma cell line LS180. Arch. Biochem. Biophys..

[21] Schinkel A.H., Wagenaar E., van Deemter L., Mol C.A., Borst P. (1995). Absence of the mdr1a P-glycoprotein in mice affects tissue distribution and pharmacokinetics of dexamethasone, digoxin, and cyclosporin A. J. Clin. Investig..

[22] Pavek P., Merino G., Wagenaar E., Bolscher E., Novotna M., Jonker J.W., Schinkel A.H. (2005). Human breast cancer resistance protein: Interactions with steroid drugs, hormones, the dietary carcinogen 2-amino-1-methyl-6-phenylimidazo(4,5-b)pyridine, and transport of cimetidine. J. Pharmacol. Exp. Ther..

[23] Schinkel A.H., Wagenaar E., Mol C.A., van Deemter L. (1996). P-Glycoprotein in the blood-brain barrier of mice influences the brain penetration and pharmacological activity of many drugs. J. Clin. Investig..

[24] König J., Cui Y., Nies A.T., Keppler D. (2000). Localization and genomic organization of a new hepatocellular organic anion transporting polypeptide. J. Biol. Chem..

[25] Köni J., Cui Y., Nies A.T., Keppler D. (2000). A novel human organic anion transporting polypeptide localized to the basolateral hepatocyte membrane. Am. J. Physiol. Gastrointest. Liver Physiol..

[26] Novotna A., Pavek P., Dvorak Z. (2011). Novel stably transfected gene reporter human hepatoma cell line for assessment of aryl hydrocarbon receptor transcriptional activity: Construction and characterization. Environ. Sci. Technol..

[27] Holló Z., Homolya L., Hegedüs T., Sarkadi B. (1996). Transport properties of the multidrug resistance-associated protein (MRP) in human tumour cells. FEBS Lett..

[28] Weiss J., Dormann S.M., Martin-Facklam M., Kerpen C.J., Ketabi-Kiyanvash N., Haefeli W.E. (2003). Inhibition of P-glycoprotein by newer antidepressants. J. Pharmacol. Exp. Ther..

[29] Weiss J., Haefeli W.E. (2006). Evaluation of inhibitory potencies for compounds inhibiting P-glycoprotein but without maximum effects: f2 values. Drug Metab. Dispos..

[30] Weiss J., Rose J., Storch C.H., Ketabi-Kiyanvash N., Sauer A., Haefeli W.E., Efferth T. (2007). Modulation of human BCRP (ABCG2) activity by anti-HIV drugs. J. Antimicrob. Chemother..

[31] Peters T., Lindenmaier H., Haefeli W.E., Weiss J. (2006). Interaction of the mitotic kinesin Eg5 inhibitor monastrol with P-glycoprotein. Naunyn Schmiedebergs Arch. Pharmacol..

[32] Albermann N., Schmitz-Winnenthal F.H., Z’graggen K., Volk C., Hoffmann M.M., Haefeli W.E., Weiss J. (2005). Expression of the drug transporters MDR1/ABCB1; MRP1/ABCC1; MRP2/ABCC2; BCRP/ABCG2; and PXR in peripheral blood mononuclear cells and their relationship with the expression in intestine and liver. Biochem. Pharmacol..

[33] König S.J., Herzog M., Theile D., Zembruski N., Haefeli W.E., Weiss J. (2010). Impact of drug transporters for the cellular resistance towards saquinavir and darunavir. J. Antimicrob. Chemother..

[34] Dvorak Z., Vrzal R., Henklova P., Jancova P., Anzenbacherova E., Maurel P., Svecova L., Pavek P., Ehrmann J., Havlik R. (2008). JNK inhibitor SP600125 is a partial agonist of human aryl hydrocarbon receptor and induces CYP1A1 and CYP1A2 genes in primary human hepatocytes. Biochem. Pharmacol..

[35] Ayed-Boussema I., Pascussi J.M., Maurel P., Bacha H., Hassen W. (2011). Zearalenone activates pregnane X receptor; constitutive androstane receptor and aryl hydrocarbon receptor and corresponding phase I target genes mRNA in primary cultures of human hepatocytes. Environ. Toxicol. Pharmacol..

[36] Vandesompele J., de Preter K., Pattyn F., Poppe B., van Roy N., de Paepe A., Speleman F. (2002). Accurate normalization of real-time quantitative RT-PCR data by geometric averaging of multiple internal control genes. Genome Biol..

[37] Weiss J., Sauer A., Divac N., Herzog M., Schwedhelm E., Böger R.H., Haefeli W.E., Benndorf R.A. (2010). Interaction of angiotensin receptor type 1 blockers with ATP-binding cassette transporters. Biopharm. Drug Dispos..

[38] Mandery K., Glaeser H., Fromm M.F. (2012). Interaction of innovative small molecule drugs used for cancer therapy with drug transporters. Br. J. Pharmacol..

[39] Sarker D., Molife R., Evans T.R., Hardie M., Marriott C., Butzberger-Zimmerli P., Morrison R., Fox J.A., Heise C., Louie S. (2008). A phase I pharmacokinetic and pharmacodynamic study of TKI258; an oral; multitargeted receptor tyrosine kinase inhibitor in patients with advanced solid tumors. Clin. Cancer Res..

[40] Kim K.B., Chesney J., Robinson D., Gardner H., Shi M.M., Kirkwood J.M. (2011). Phase I/II and pharmacodynamic study of dovitinib (TKI258); an inhibitor of fibroblast growth factor receptors and VEGF receptors; in patients with advanced melanoma. Clin. Cancer Res..

[41] Xiao Z., Ding N., Xiao G., Wang S., Wu Y., Tang L. (2012). Reversal of multidrug resistance by gefitinib via RAF1/ERK pathway in pancreatic cancer cell line. Anat. Rec..

[42] Garcia R., Franklin R.A., McCubrey J.A. (2006). EGF induces cell motility and multi-drug resistance gene expression in breast cancer cells. Cell Cycle.

[43] McCubrey J.A., Steelman L.S., Abrams S.L., Lee J.T., Chang F., Bertrand F.E., Navolanic P.M., Terrian D.M., Franklin R.A., D’Assoro A.B. (2006). Roles of the RAF/MEK/ERK and PI3K/PTEN/AKT pathways in malignant transformation and drug resistance. Adv. Enzyme Regul..

[44] Yang H.Y., Zhao L., Yang Z., Zhao Q., Qiang L., Ha J., Li Z.Y., You Q.D., Guo Q.L. (2012). Oroxylin A reverses multi-drug resistance of human hepatoma BEL7402/5-FU cells via downregulation of P-glycoprotein expression by inhibiting NF-κB signaling pathway. Mol. Carcinog..

[45] Yueh M.F., Mellon P.L., Tukey R.H. (2011). Inhibition of human UGT2B7 gene expression in transgenic mice by the constitutive androstane receptor. Mol. Pharmacol..

[46] Harmsen S., Meijerman I., Maas-Bakker R.F., Beijnen J.H., Schellens J.H. (2013). PXR-mediated P-glycoprotein induction by small molecule tyrosine kinase inhibitors. Eur. J. Pharm. Sci..

[47] Ebert B., Seidel A., Lampen A. (2005). Identification of BCRP as transporter of benzo(a)pyrene conjugates metabolically formed in Caco-2 cells and its induction by Ah-receptor agonists. Carcinogenesis.

[48] Zhang L., Zhang Y.D., Strong J.M., Reynolds K.S., Huang S.M. (2008). A regulatory viewpoint on transporter-based drug interactions. Xenobiotica.

[49] Pfrunder A., Gutmann H., Beglinger C., Drewe J. (2003). Gene expression of CYP3A4; ABC-transporters (MDR1 and MRP1-MRP5) and hPXR in three different human colon carcinoma cell lines. J. Pharm. Pharmacol..

[50] Kliewer S.A., Goodwin B., Willson T.M. (2002). The nuclear pregnane X receptor: A key regulator of xenobiotic metabolism. Endocr. Rev..

[51] Urquhart B.L., Tirona R.G., Kim R.B. (2007). Nuclear receptors and the regulation of drug-metabolizing enzymes and drug transporters: Implications for interindividual variability in response to drugs. J. Clin. Pharmacol..

[52] Jigorel E., Le Vee M., Boursier-Neyret C., Parmentier Y., Fardel O. (2006). Differential regulation of sinusoidal and canalicular hepatic drug transporter expression by xenobiotics activating drug-sensing receptors in primary human hepatocytes. Drug Metab. Dispos..

[53] Teng S., Jekerle V., Piquette-Miller M. (2003). Induction of ABCC3 (MRP3) by pregnane X receptor activators. Drug Metab. Dispos..

[54] Kast H.R., Goodwin B., Tarr P.T., Jones S.A., Anisfeld A.M., Stoltz C.M., Tontonoz P., Kliewer S., Willson T.M., Edwards P.A. (2002). Regulation of multidrug resistance-associated protein 2 (ABCC2) by the nuclear receptors pregnane X receptor; farnesoid X-activated receptor; and constitutive androstane receptor. J. Biol. Chem..

[55] Geick A., Eichelbaum M., Burk O. (2001). Nuclear receptor response elements mediate induction of intestinal MDR1 by rifampin. J. Biol. Chem..

[56] Hariparsad N., Nallani S.C., Sane R.S., Buckley D.J., Buckley A.R., Desai P.B. (2004). Induction of CYP3A4 by efavirenz in primary human hepatocytes: Comparison with rifampin and phenobarbital. J. Clin. Pharmacol..

